# Ultrasonographic findings in goats with contagious caprine pleuropneumonia caused by *Mycoplasma capricolum* subsp. *capripneumoniae*

**DOI:** 10.1186/s12917-017-1167-4

**Published:** 2017-08-22

**Authors:** Mohamed Tharwat, Fahd Al-Sobayil

**Affiliations:** Department of Veterinary Medicine, College of Agriculture and Veterinary Medicine, Qassim University, Buraydah, Saudi Arabia

**Keywords:** Contagious caprine pleuropneumonia, CCPP, Goat, Mycoplasma, Ultrasonography

## Abstract

**Background:**

In goats, contagious caprine pleuropneumonia (CCPP) is a cause of major economic losses in Africa, Asia and in the Middle East. There is no information emphasising the importance of diagnostic ultrasound in goats with CCPP caused by *Mycoplasma capricolum* subsp. *capripneumoniae* (*Mccp*). This study was designed to describe the ultrasonographic findings in goats with CCPP caused by *Mccp* and to correlate ultrasonographic with post-mortem findings. To this end, 55 goats with CCPP were examined. Twenty-five healthy adult goats were used as a control group.

**Results:**

Major clinical findings included harried, painful respiration, dyspnoea and mouth breathing. On ultrasonography, a liver-like echotexture was imaged in 13 goats. Upon post-mortem examination, all 13 goats exhibited unilateral pulmonary consolidation. Seven goats had a unilateral hypoechoic pleural effusion. At necropsy, the related lung was consolidated and the pleural fluid appeared turbid and greenish. Pleural abscessiation detected in five goats was confirmed post-mortem. Twenty-eight goats had a bright, fibrinous matrix extending over the chest wall containing numerous anechoic fluid pockets with medial displacement and compression of lung tissue. Echogenic tags imaged floating in the fluid were found upon post-mortem examination to be fibrin. In two goats, a consolidated right parenchyma was imaged together with hypoechoic pericardial effusions with echogenic tags covering the epicardium. At necropsy, the right lung was consolidated in three goats and fibrin threads were found covering the epicardium and pericardium.

**Conclusions:**

In goats with CCPP, the extension and the severity of the pulmonary changes could not be verified with clinical certainty in most cases, whereas this was possible most of the time with sonography, thus making the prognosis easier. Ultrasonographic examination of the pleurae and the lungs helped in the detection of various lesions.

## Background

In many parts of the world, goats are considered important domestic animals. They are kept as a source of meat, milk, cheese, and fibre. Goats are also wonderful to raise purely for enjoyment, as a hobby or for show. Goats can survive in harsh environments in which other livestock species would perish. In addition, they are able to live and reproduce in icy mountainous areas as well as in the hot, dry desert. Therefore, improved goat husbandry will help maximize human food supplies from marginal agricultural lands under restrictive climatologic circumstances [1].

Among the important goat diseases, mycoplasmal infections result in significant losses in many countries, and morbidity and mortality can reach 100% [1]. Of these mycoplasmal infections, contagious caprine pleuropneumonia (CCPP), occurring in many countries in Asia and Africa, is a severe contagious respiratory disease of goats [2]. It is characterised by fever, high morbidity, and high mortality. Respiration is accelerated and painful, coughing is frequent, and, in the terminal stages, the animal is unable to move, standing base wide with neck extended. The gross lesions of the disease are typically limited to the thoracic cavity and characterised by fibrinous pleuropneumonia, lung hepatisation, and accumulation of pleural fluid [3–5]. The pneumonia may often be unilateral [3, 6]. Rapid and inexpensive detection of CCPP is carried out using a *Mccp* capsular polysaccharide-specific antigen detection latex agglutination test (LAT) [7–10]. The World Organization for Animal Health (OIE) has recommended the LAT for confirmation of clinical cases of goats with CCPP [2].

The type, severity, and extent of lung disease cannot always be determined by physical examination alone, which may lead to misinterpretation of respiratory symptoms and ineffective therapy [11]. Because the waves are incapable of penetrating gas-filled structures, physiologically normal lung tissue cannot be examined by ultrasound; however, sonography is suitable for the detection of a number of respiratory pathologies [12–17].

In sheep, accurate identification and distribution of pleural and superficial lung pathology necessitate ultrasonographic examination. Ultrasonographic examination of the chest allows critical evaluation of the pleurae and establishment of a definitive diagnosis in most diseased sheep [18–21]. In goats, however, only one report of thoracic osteosarcoma was found in the veterinary literature [22]. The present study was designed to describe the ultrasonographic findings in goats with CCPP. The clinical and post-mortem findings are also described.

## Methods

### Animals, history and physical examination

Fifty-five goats (mean age 2.5 ± 1.1 years; mean body weight 26.4 ± 10.1 kg) were examined in the Veterinary Teaching Hospital, Qassim University, Saudi Arabia, between February 2010 and August 2015. The goats had been admitted because of weight loss, anorexia and respiratory signs which included dyspnoea, polypnea, cough and nasal discharges. Twenty-five healthy adult goats (mean age 2.8 ± 0.9 years; mean body weight 31.0 ± 12.7 kg) were used as a control group.

The diseased goats were enrolled in the study in situ based on a positive serological LAT (CapriLAT, product code: RAI 6224, lot number: MccpLAT304141, Animal Health and Veterinary Laboratories Agency, Surrey, United Kingdom) that confirmed the detection of *Mccp* as the causative agent of CCPP [7–10]. The control goats were enrolled based on a negative result of the LAT. The owners of the goats (both sick and controls) provided informed consent for their animals to participate in the study, and the owners of the controls gave permission for the healthy animals to be euthanized.

### Ultrasonographic examination

A real-time, B-mode ultrasound machine equipped with a 7.5 MHz-sector transducer (SSD-500, Aloka, Tokyo, Japan) was used to image the thorax and heart in the non-sedated diseased goats. Parallel, the lungs and the heart was scanned in the control animals. Firstly, the two sides of the thorax of each animal were clipped and the skin was shaved. The thoracic ultrasonography was carried out in the goats as in that reported for sheep [19, 20], and the echocardiography was conducted as has been recently reported [23]. As reported by Buczinski et al. [24], criteria for lung consolidation, pleural fluid accumulation, fibrinous pleurisy and pericardial fluid accumulation was defined as follows. Lung consolidation was defined as the ability to observe the abnormal lung parenchyma as a heterogenous hypoechoic to echoic area. Pleural fluid accumulation was diagnosed if disruptions between the parietal and the visceral pleura were observed during examination. Fibrinous pleurisy was defined if fibrinous matrix was observed during examination extending over the chest wall and containing numerous fluid pockets, and the pleural line was serrated with an irregular shape. Pericardial fluid accumulation was diagnosed if disruptions between the parietal and the visceral pericardium were observed during examination.

### Postmortem examination

Both the diseased and control goats were euthanized by throat cutting without breaking the neck and thoroughly examined post-mortem. If present, the lung consolidation, pleural fluid accumulation, fibrinous pleurisy, pericardial fluid accumulation were recorded and described.

## Results

Of the 47 females and eight males, 45 were local goat breeds (Ardi) and the remaining 10 were Syrian goats. On initial examination, the diseased goats had a mean internal body temperature of 39.8 ± 1.7 °C, a mean pulse of 122 ± 23 beats per minute and a mean respiratory rate of 45 ± 9 breaths per minute. Harried, painful respiration was detected in 48 goats, 15 had dyspnoea and 33 animals displayed open mouth breathing. Spontaneous coughing was detected in 39 goats and seven had coughing upon stimulation. Eighteen goats were admitted in a recumbent position. Surprisingly, nine goats had no history of coughing at all and did not cough upon stimulation. Fifty-one of the 55 diseased goats were admitted in a depressed and poor body condition. Upon percussion of the lungs, 51 goats exhibited a unilaterally reduced volume and four had increased resonance. Upon auscultation of the lungs, 24 cases exhibited unilaterally mild to severe increased vesicular breath sounds, nine had rough breath sounds, nine had splashing and pleuritic friction sounds, six had an absence of lung sounds, and wheezing was detected in seven cases. Some goats had a combination of these findings. In all 55 diseased goats, the LAT was positive for the presence of *Mccp*.

Imaging of the lungs in the control goats revealed normally aerated lungs characterised by the uppermost hyperechoic linear image with numerous reverberation artefacts running regularly and parallel below this line. Both pleural leaves appeared as a broad, smooth, hyperechoic line between the surface of the lungs and the musculature of the thoracic wall moving synchronously with respiration. It was not possible to differentiate the parietal and visceral pleurae. The motion of the lungs synchronous with respiration was visible. No pleural fluid was visualised in any of the control goats. Upon post-mortem examination, none of the control goats displayed any pulmonary abnormality.

**Fig. 1:**
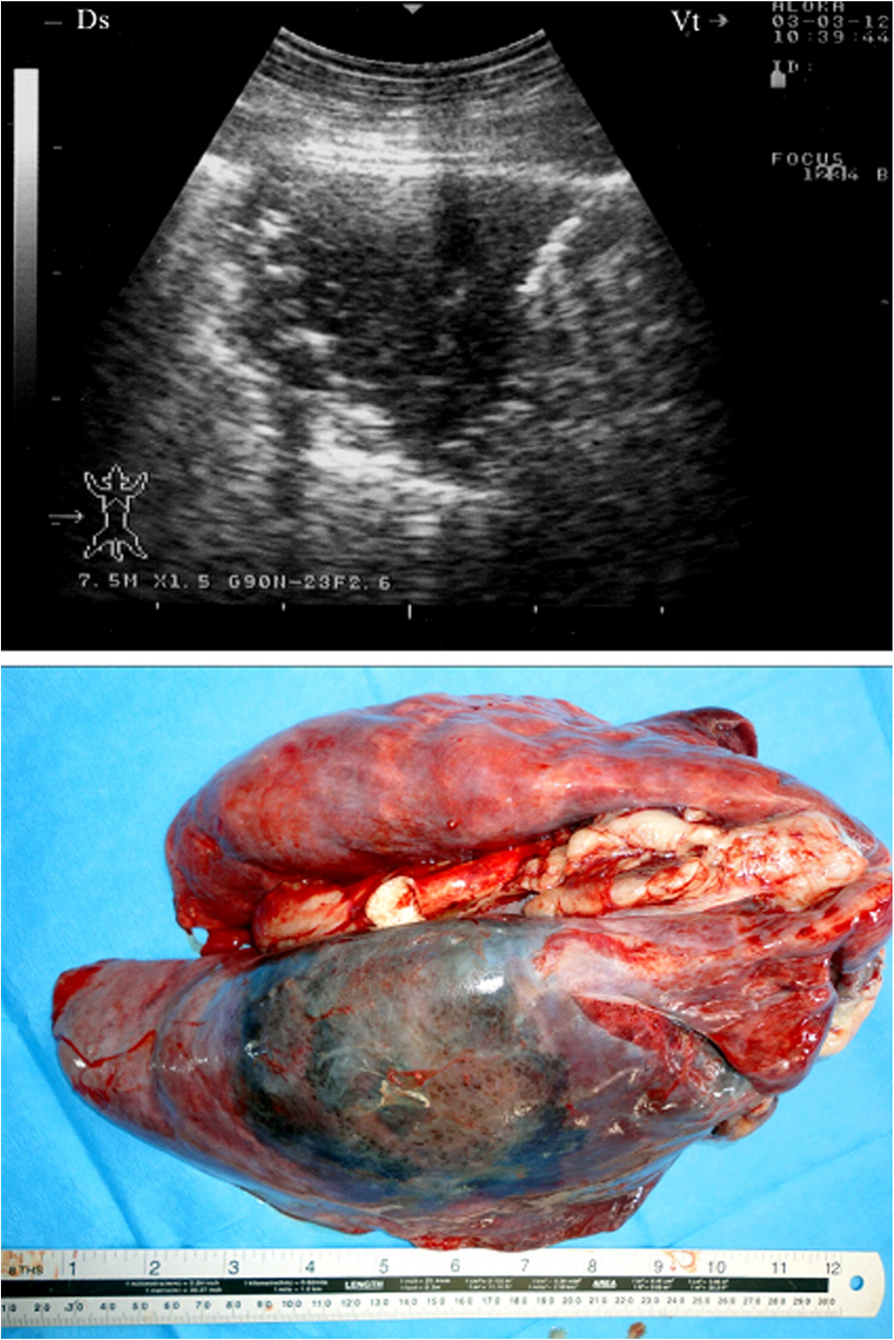
Liver-like hypoechoic echotexture in a goat with *Mccp-*caused CCPP. Pulmonary consolidation was detected post-mortem

**Fig. 2:**
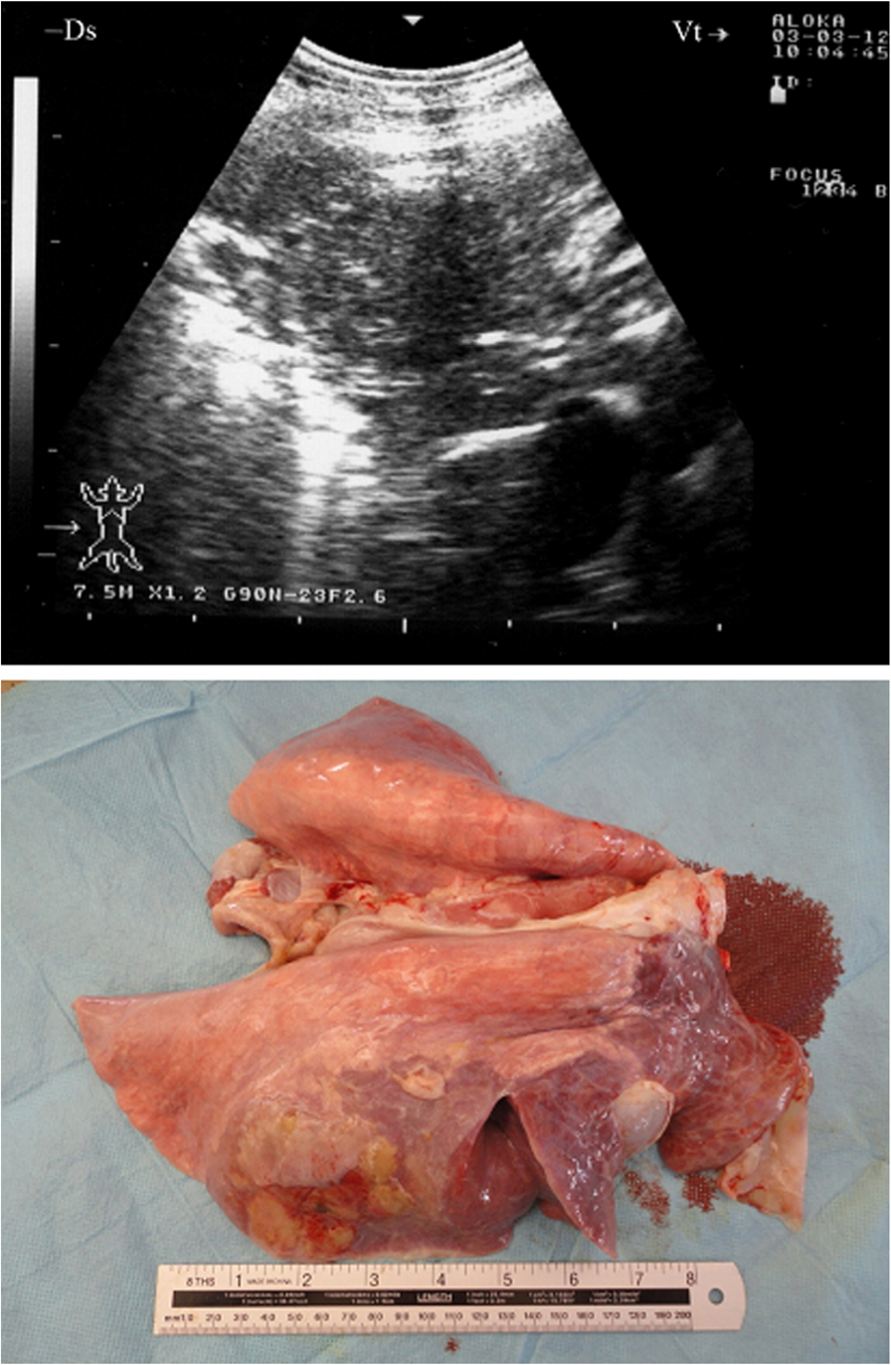
Liver-like hypoechoic echotexture in a goat with *Mccp-*caused CCPP. Pulmonary consolidation was detected post-mortem

In 13 of the diseased goats, a non-ventilated lung parenchyma with a liver-like echotexture was imaged. Depending on the degree of atelectasis, the ventilated lung deep to the consolidation could be identified by the weak, defined, and blurry reverberation artefacts. The extensive hypoechoic zones in the cranioventral lung fields and the cranioventral portions of the main lobes were confirmed to be consolidated lung tissue upon post-mortem examination (Figs. 1 and 2). The post-mortem examination showed all 13 goats to have unilateral pulmonary consolidation: nine at the diaphragmatic lobes (Fig. 1) and four at the diaphragmatic together with the anterior lobes (Fig. 2). Remarkably, 11 goats had consolidation in the right lung and only two in the left lung.

**Fig. 3:**
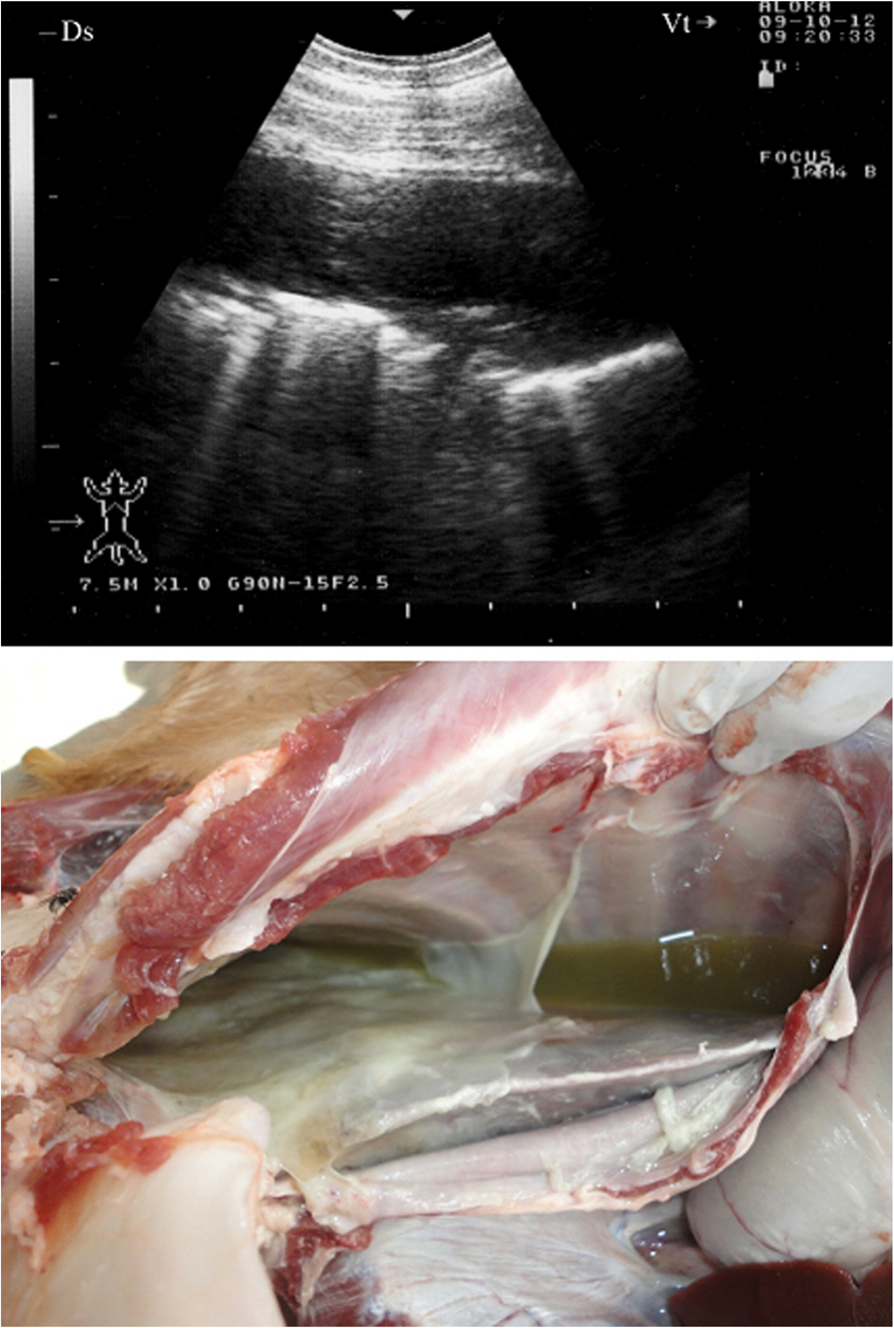
Unilateral hypoechoic pleural effusion in CCPP goat. Necropsy revealed consolidated lung and *greenish pleural fluid*

**Fig. 4:**
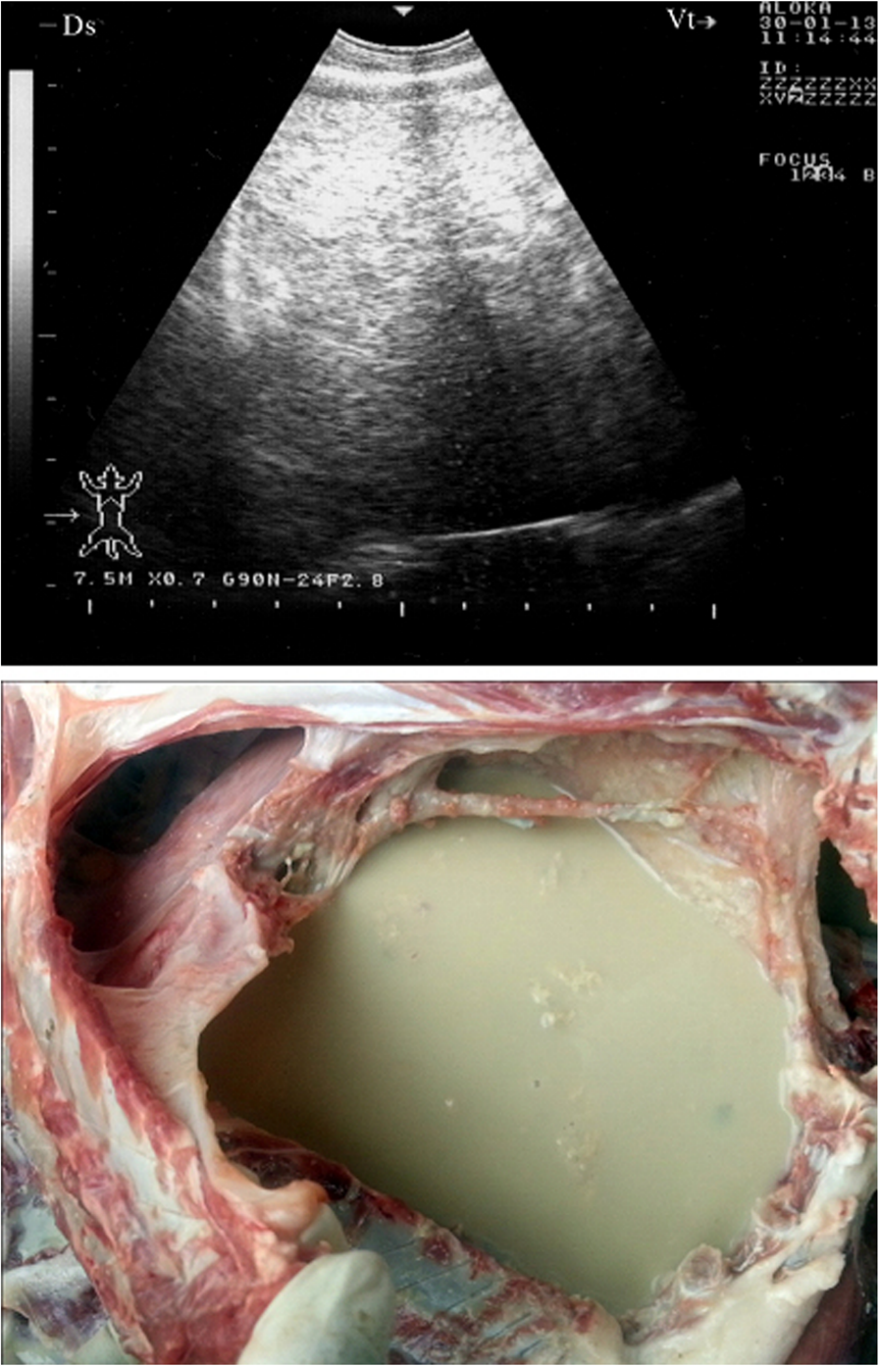
Pleural abscessation in a goat with CCPP caused by *Mccp*. Diagnosis was confirmed post-mortem

Ultrasonography of the thorax in seven goats revealed a unilateral pleural effusion that appeared hypoechoic (Fig. 3). The visceral pleura appeared broader and more hyperechoic than normal due to acoustic enhancement by the pleural exudates. Upon necropsy, the related lung was consolidated and the pleural fluid appeared turbid and greenish. Five other goats displayed a free hypo-and anechoic fluid with echogenic foci. At post-mortem examination, it was confirmed to be pus (Fig. 4).

**Fig. 5:**
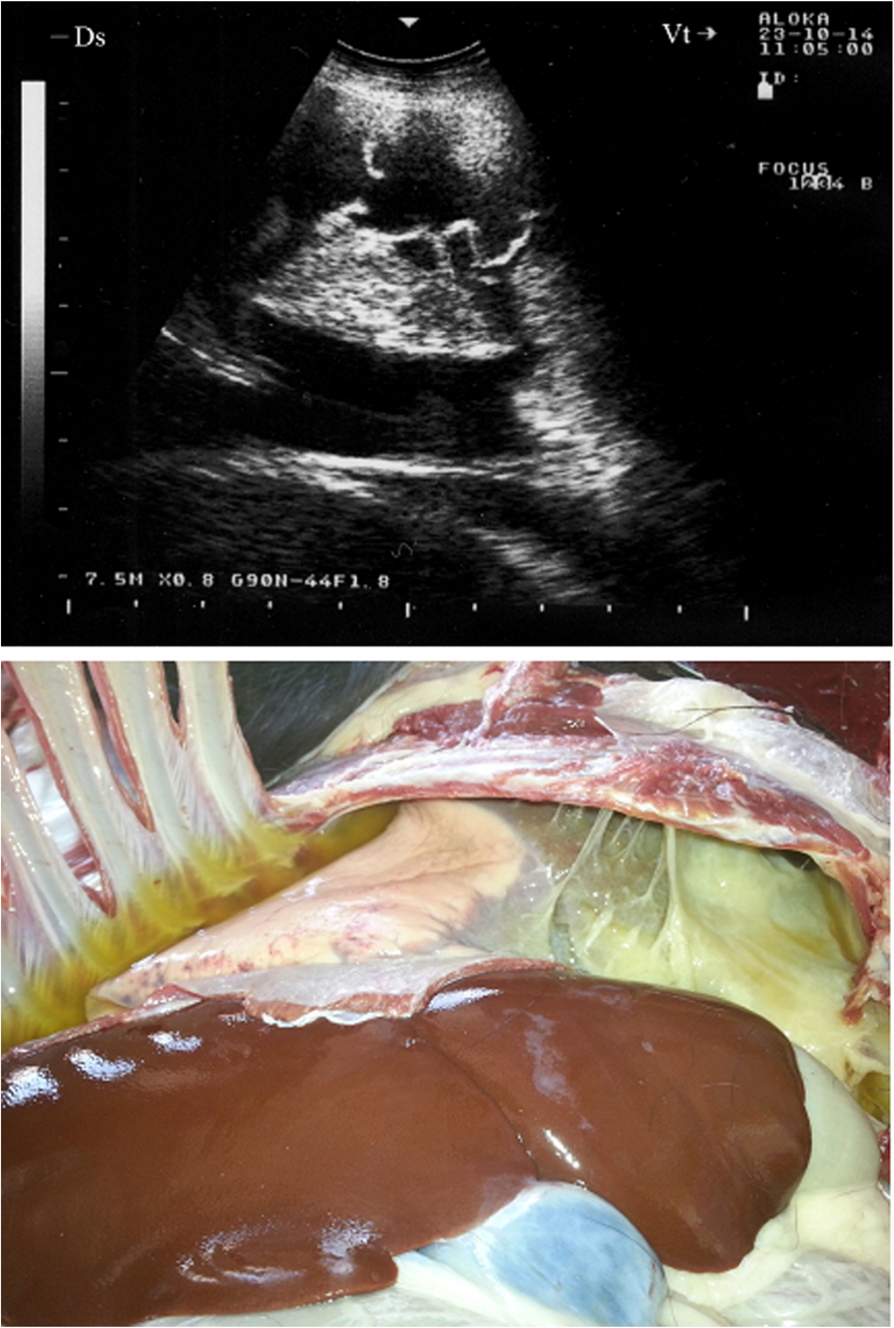
Marked fibrinous pleurisy in a goat with *Mccp*-caused CCPP. Necropsy revealed *clear yellow pleural fluid*

**Fig. 6:**
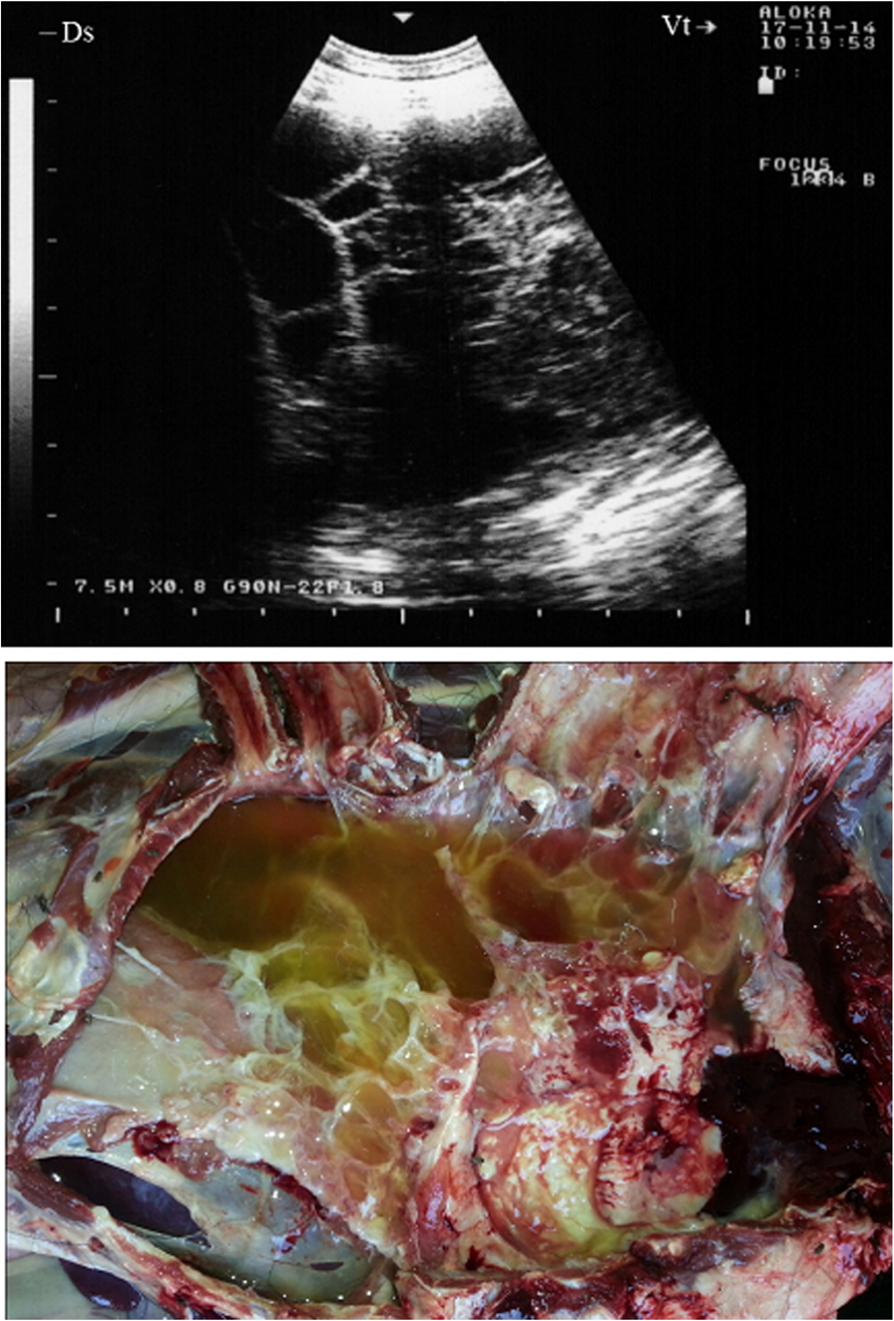
Marked fibrinous pleurisy in a goat with *Mccp*-caused CCPP. Necropsy revealed turbid *yellowish pleural fluid*

**Fig. 7:**
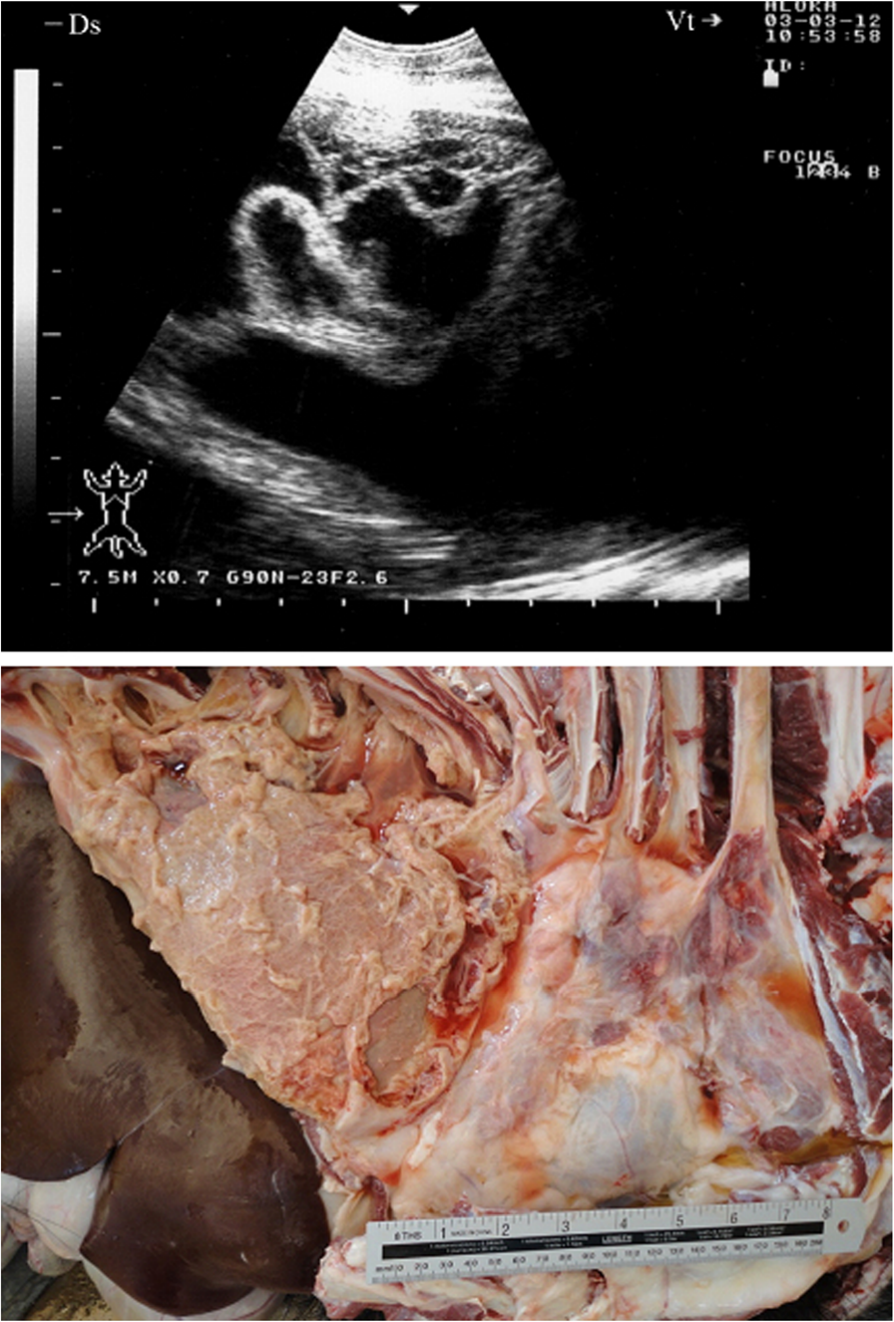
Marked fibrinous pleurisy in a goat with *Mccp*-caused CCPP. Necropsy revealed *reddish pleural fluid*

**Fig. 8:**
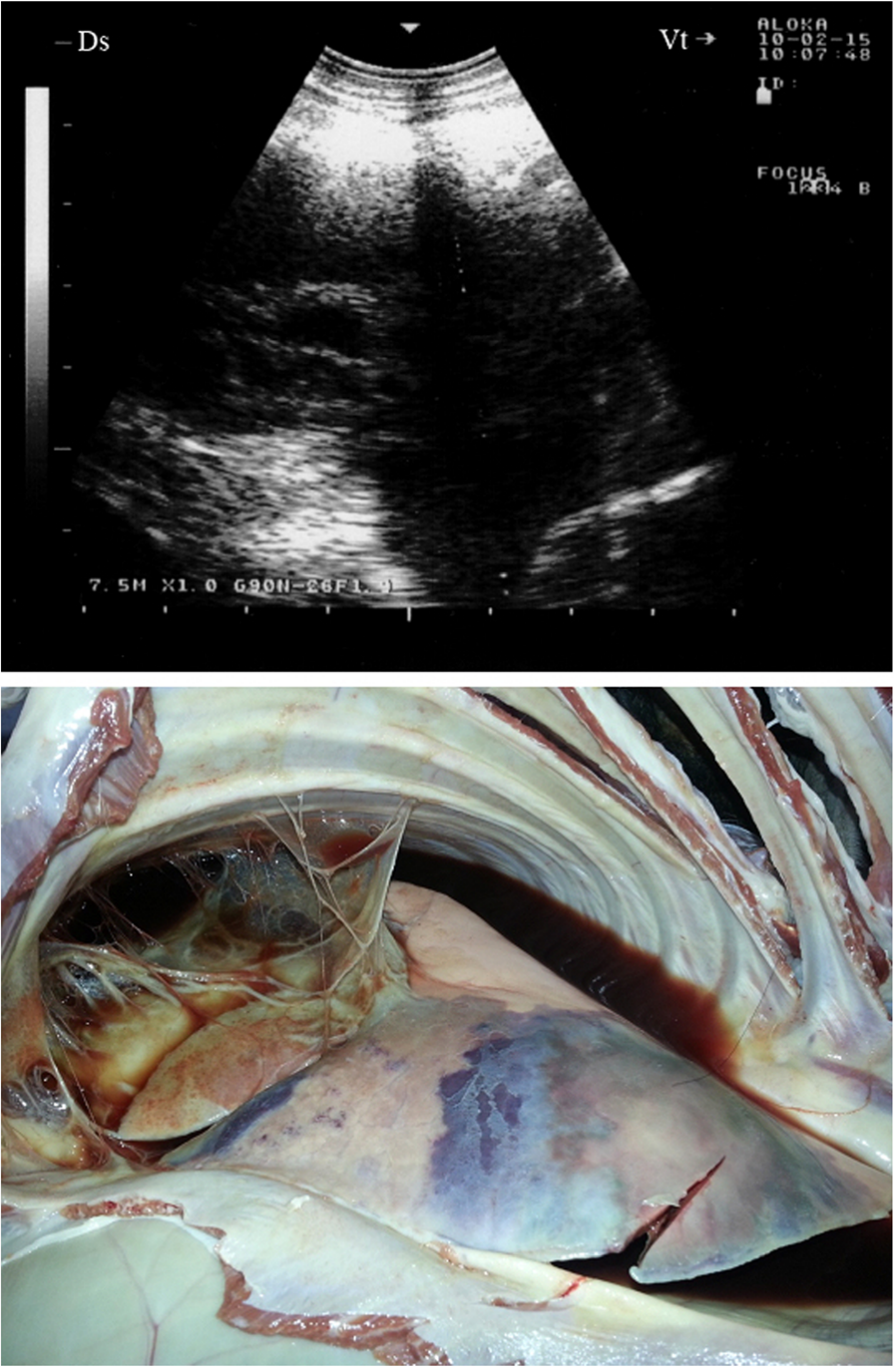
Marked fibrinous pleurisy in a goat with *Mccp*-caused CCPP. Necropsy revealed *dark red pleural fluid*

**Fig. 9:**
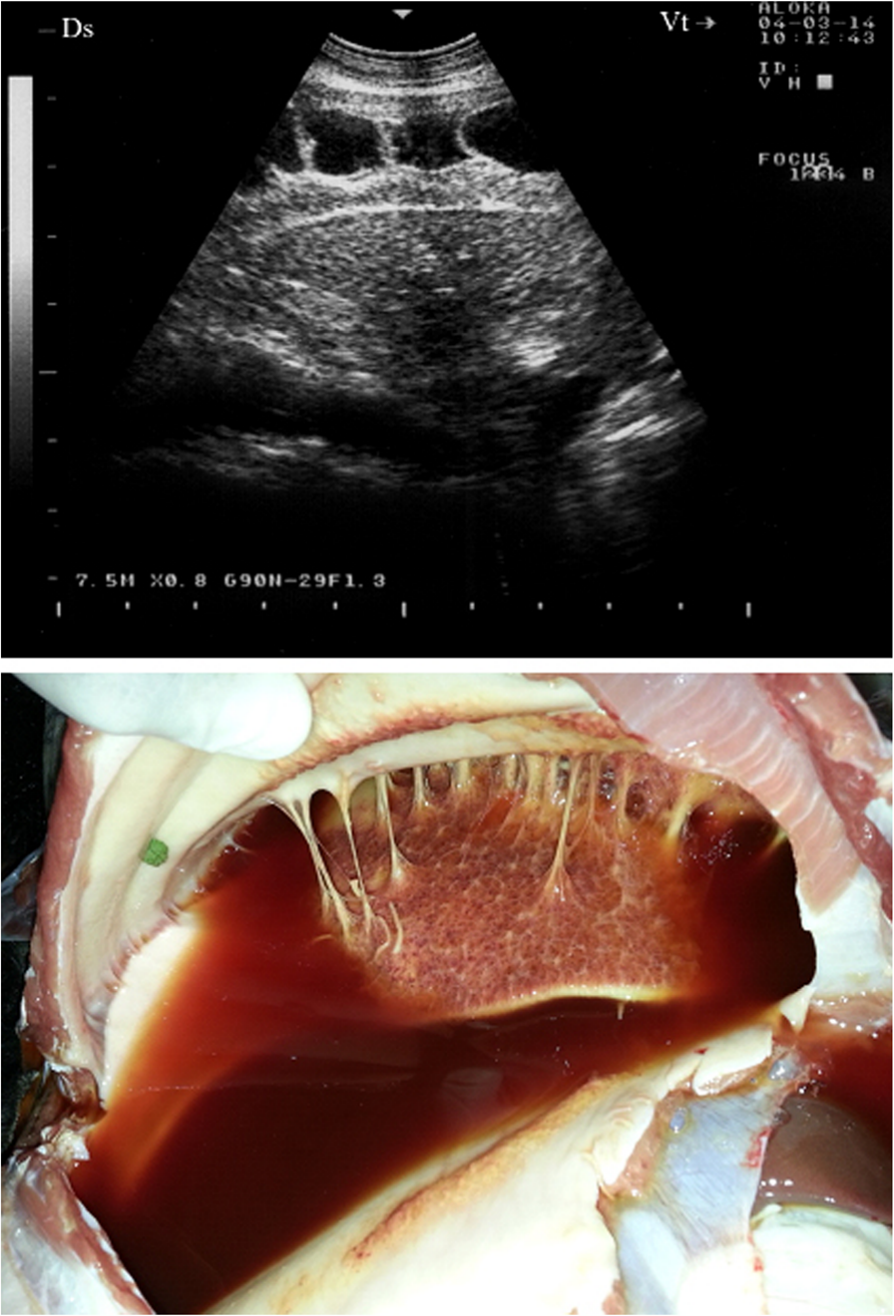
Marked fibrinous pleurisy in a goat with *Mccp*-caused CCPP. Necropsy revealed *dark red pleural fluid*

Sonograms obtained from 28 goats with marked fibrinous pleurisy revealed a bright fibrinous matrix extending over the chest wall containing numerous anechoic fluid pockets with medial displacement and compression of lung tissue (Figs. 5, 6, 7, 8, 9). Due to acoustic enhancement, the surface of the displaced lung lobes had the appearance of broad hyperechoic lines. Echogenic tags that were imaged floating in the fluid were found to be fibrin upon post-mortem examination. The pleural fluid was a clear yellow in eight goats (Fig. 5), turbid and yellowish in five (Fig. 6), reddish in eight (Fig. 7) and dark red in the remaining seven goats (Figs. 8 and 9).

**Fig. 10:**
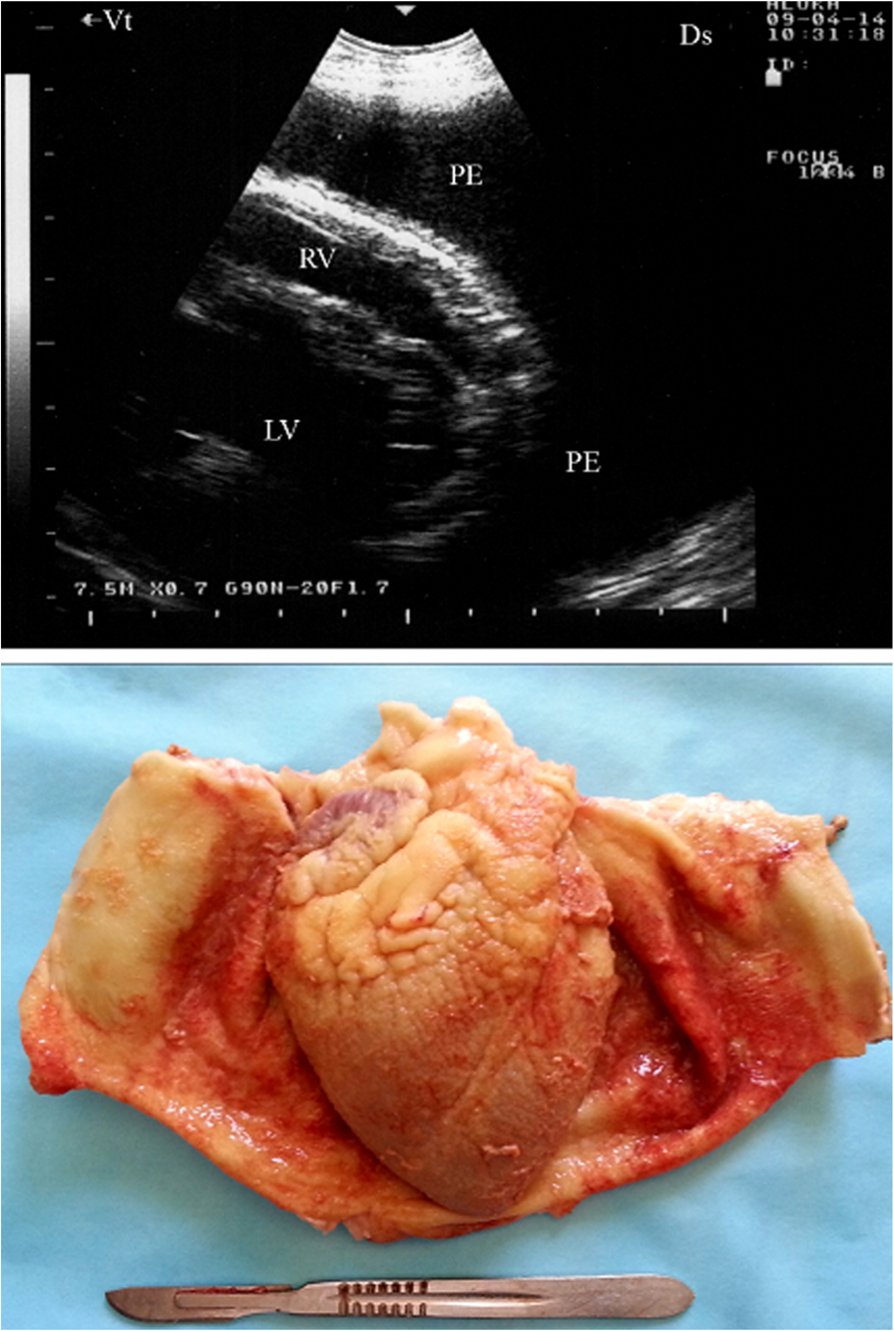
Pericardial effusions and echogenic tags of epicardium in CCPP goat. Necropsy showed consolidated right lung

In two goats, a consolidated right parenchyma was imaged together with hypoechoic pericardial effusions and echogenic tags covering the epicardium (Fig. 10). Upon necropsy, the right lung was consolidated in three goats and fibrin threads were found covering the epicardium and pericardium.

## Discussion

To the author’s knowledge, there is no information emphasising the importance of diagnostic ultrasound in goats with CCPP caused by *Mccp*. A knowledge of the pathological lung changes is necessary in order to evaluate the diagnostic value of ultrasonography. In this study, post-mortem examinations were therefore undertaken to evaluate the diagnostic value of the imaging technique.

In human medicine, conventional radiographs are still the first diagnostic imaging choice for thoracic examination [25]. Similarly, in veterinary medicine, radiography is superior to ultrasonography in the identification of diffuse diseases of lung parenchyma such as pulmonary emphysema, oedema, interstitial pneumonia and diffuse neoplastic or granulomatous processes [26–28]. However, unlike radiography, ultrasonography requires no special restrictions or health and safety procedures [19].

As shown in the present study, in a healthy animal, pulmonary air content complicates the ultrasonographic assessment of lung parenchyma. Due to total reflection, the intercostal transmission of ultrasound where the lungs are air-filled extends only to the visceral pleura and ends at the air-filled alveoli [29]. A useful ultrasonographic assessment of the lung tissue can be achieved when the pulmonary air content is reduced and the lung has the appearance of liver. The irregularity of the visceral pleural surface can be a first sign of consolidation [28].

In the consolidated lung areas in cattle and horses, there are still varying numbers of air-filled alveoli that form hyperechoic zones [12, 30, 31]. In correlation to the size of the compressed lung area and the duration of the existence of a consolidation, a reduction of these hyperechoic zones can be detected as hyperechoic reflective bands [12, 32, 33]. In calves, Rabeling et al. [34] have reported consolidations as echogenic regions with comet-tail artefacts. In the present study, the consolidations were always hypoechoic and homogenous, possibly due to the accumulation of exudate, blood and mucous. Similar findings have been reported previously [16, 30]. In horses, consolidation was observed most often cranioventrally, whereby the right lung was usually more severely affected [30]. In this study, consolidation was observed mostly caudodorsally in the right lung.

In bovines, the most profound lung changes can be observed in most cases in the cranial and above all the cranioventral lung areas. Therefore, it is advisable to begin the sonographic examination in that location [31]. In contrast, in this study, most lung changes were detected in the caudodorsal lung areas.

In this study, ultrasonography allowed the pleural effusion to be visualised in a much more definitive manner and was able to qualify the nature and the extent of the effusion. Pleural effusion appears as an anechoic space between the lung, thoracic wall, diaphragm and heart, with acoustic enhancement deep to the lesion and often with septa floating within it [28]. The parital and visceral pleurae were also separated from one another; similar findings were reported by Reef et al., [26]. Within the pleural cavity, anechoic fluid represents transudate, and increased echogenicity points toward an increased cell count or total protein concentration [12, 26, 28]. This feature is, however, unreliable and must always be confirmed by thoracocentesis [35].

In practice, ultrasonography is mostly used for diagnostic purposes in the case of pleural effusion. The images are often characteristic, and ultrasound-guided aspiration provides a safe way to obtain liquid for analysis. Another use for such technology is the identification of the area to be drained so as to provide relief to the patient. It is also an objective tool for monitoring patients secondary to therapy [16]. Ultrasonography has been most helpful in the definitive diagnosis of superficial lung abscesses where the anechoic areas containing multiple hyperechoic dots were bordered distally by a broad hyperechoic capsule [21]. The present ultrasonographic findings of pleural abscesses were similar to the ultrasonographic description of suppurative pleuropneumonia in a ram [35–37].

This study has 2 limitations; first was using the LAT in detecting goats with CCPP. A conclusive diagnosis should have included culture or a molecular confirmation of the pathogen. Excluding co-infections with bacteriology was a second limitation. We aimed to assist filed veterinarians in early detection and isolation of suspected cases until the results of culture or molecular confirmation arrive. However, the causative agent, *Mccp*, is very difficult to cultivate in vitro. This may be attributed to its fastidiousness and/or misuse of antimicrobials [38]. In addition, current serological tests used for detection of *Mccp* antibodies include the growth inhibition test, complement fixation test (CFT), indirect haemagglutination assay, competitive enzyme-linked immunosorbent assay and LAT (c-ELISA). The first two tests are ranked the least sensitive for confirmation of clinical CCPP cases (+); the third and fourth are suitable (++), and the fifth (+++) is the recommended [2]. The LAT is considered the simplest, quick and excellent procedure for the “*in field* or in situ” diagnosis of CCPP either in whole blood or serum [39]. Moreover, the LAT is reported to be more sensitive than the CFT [40] and also easier to perform than the c-ELISA [41].

## Conclusions

In this study, some ultrasound images in goats with CCPP were characteristic of pathologic lesions in the chest and can help clinicians with their diagnosis by allowing visualisation of the lesion itself or serving as a guide for aspiration. In current veterinary farm practice, however, in which radiographic examination is impossible, ultrasonography is an available diagnostic tool that is quickly implemented and non-invasive.
